# A novel strategy for eradication of staphylococcal biofilms using blood clots

**DOI:** 10.3389/fcimb.2025.1507486

**Published:** 2025-01-31

**Authors:** Kayla E. Grooters, Sheridan L. Hayes, David M. Richter, Jennifer C. Ku, Robert Sawyer, Yong Li

**Affiliations:** ^1^ Department of Medicine, Western Michigan University Homer Stryker M.D. School of Medicine, Kalamazoo, MI, United States; ^2^ Division of Medical Engineering, Department of Surgical Science, Western Michigan University Homer Stryker MD School of Medicine, Kalamazoo, MI, United States

**Keywords:** biofilm, antibiotic resistance, staphylococci, prosthetic joint infections, biomaterials

## Abstract

**Introduction:**

Infections with coagulase negative staphylococcal species (CoNS) are a major cause of mortality and morbidity in joint and heart valve replacement procedures, largely due to biofilm formation. Cells within biofilms have higher rates of antibiotic resistance than their planktonic counterparts; consequently, novel mechanisms are needed to combat these infections.

**Methods:**

To enhance antibiotic delivery and penetration, this innovative study involved treating CoNS biofilms with murine blood clots impregnated with antibiotics. We then investigated the impact of this treatment on biofilm density, metabolism, and architecture.

**Results:**

Our pilot study demonstrates that this method of antibiotic delivery results in improved biofilm clearance, relative to conventional exposure methods.

**Discussion:**

Our results demonstrate that blood clot exposure has an intrinsic impact on biofilm density and potentially reduces colonization, warrenting further investigation into the mechanism.

## 1 Introduction

A biofilm is a three-dimensional structure consisting of microscopic organisms and a robust extracellular matrix (ECM) produced by such organisms. This ECM functions to enhance adhesion to the local environment, reduce desiccation, mitigate environmental hazards, and provide protection from antimicrobial compounds. Accordingly, these biofilms become highly resistant to antibiotics (Sharma et al., 2019). Biofilm infections are a major cause of morbidity and mortality—particularly impacting prosthetic joints and valves. Prosthetic medical implants are susceptible to colonization by microorganisms during the perioperative and postoperative period (Khatoon et al., 2018; Skovdal et al., 2022). Infections during the perioperative period are most commonly due to inoculation of microorganisms during the surgery due to a lapse in sterile technique. Rarely, inoculation can occur during the manufacturing process (Ribeiro et al., 2012). Postoperative infections occur when the implant is seeded by microorganisms after the initial implantation. These microorganisms can be introduced through hematogenous or contiguous routes. Infections can further be classified as early, which occur within 3 months of the operation, delayed, which occur within 3-24 months, and late, occurring more than 24 months postoperatively (Ribeiro et al., 2012).

Staphylococci, including the coagulase negative *Staphylococcus epidermidis*, are the most prevalent biofilm-producing species (Oliveira et al., 2018; Otto, 2008, 2018). Staphylococcal species are responsible for over two thirds of implantable device associated infections (Darouiche, 2004; Oliveira et al., 2018; Severn and Horswill, 2023). These gram-positive bacteria are part of the naturally occurring skin and soft tissue microbiota in humans, and therefore can easily contaminate the surgical field. (Khatoon et al., 2018; Severn and Horswill, 2023). Biofilm formation can start within 24 h of inoculation. These infections are often refractory to antibiotic treatment and require surgical revision (Skovdal et al., 2022). The annual cost of revision surgery due to biofilm-mediated infections is $7.849 million globally (Cámara et al., 2022).

One of the key issues with using antibiotics to treat biofilms is achieving the required minimum bactericidal concentration (MBC) of a drug at the infection site. The MBC for a biofilm can be thousands of times greater than the MBC for planktonic cells (Oliveira et al., 2018). This is because the ECM provides a physical barrier which protects cells from antibiotic exposure. In addition, there exists a stratification of metabolic activity through the layers of the biofilm (Shree et al., 2023; Wood et al., 2013). The metabolically inactive basement cells form the foundation for the biofilm and can persist to reform the biofilm despite eradication of the upper layers (Wood et al., 2013). For these reasons, there is a growing need to develop new technology capable of penetrating and eradicating biofilm infections. One idea is to use a biologic delivery system that can adhere to the biofilm itself, allowing direct exposure and penetration of the drug. Previous studies have implemented fibrin clots as substrates to model biofilms, indicating that they are capable of biofilm integration (Domínguez-Herrera et al., 2012). Our team elected to suspend antibiotics within a blood clot and then adhere it to the biofilm. Our recent studies have shown that these blood clots can release a steady concentration of antibiotics into the target environment for at least 7 days (Ku et al., 2024). We hypothesized that this Trojan horse model would facilitate penetration of the antibiotic throughout the biofilm, leading to an increased rate of clearance compared to conventional treatments.

## 2 Materials and methods

### 2.1 Chemicals, media, and strains

*Staphylococcus epidermidis* FDA strain PCI 1200 (ATCC Cat#12228) was routinely maintained as a frozen –80°C stock and grown on Luria–Bertani (LB) agar (Fisher Scientific). Gentamicin (Gibco; 10 µg/mL in LB broth) and vancomycin (Thermofisher; 20 µg/mL in LB broth) were suspended in LB broth for inhibition experiments.

### 2.2 Biofilm formation

**Figure 1:**
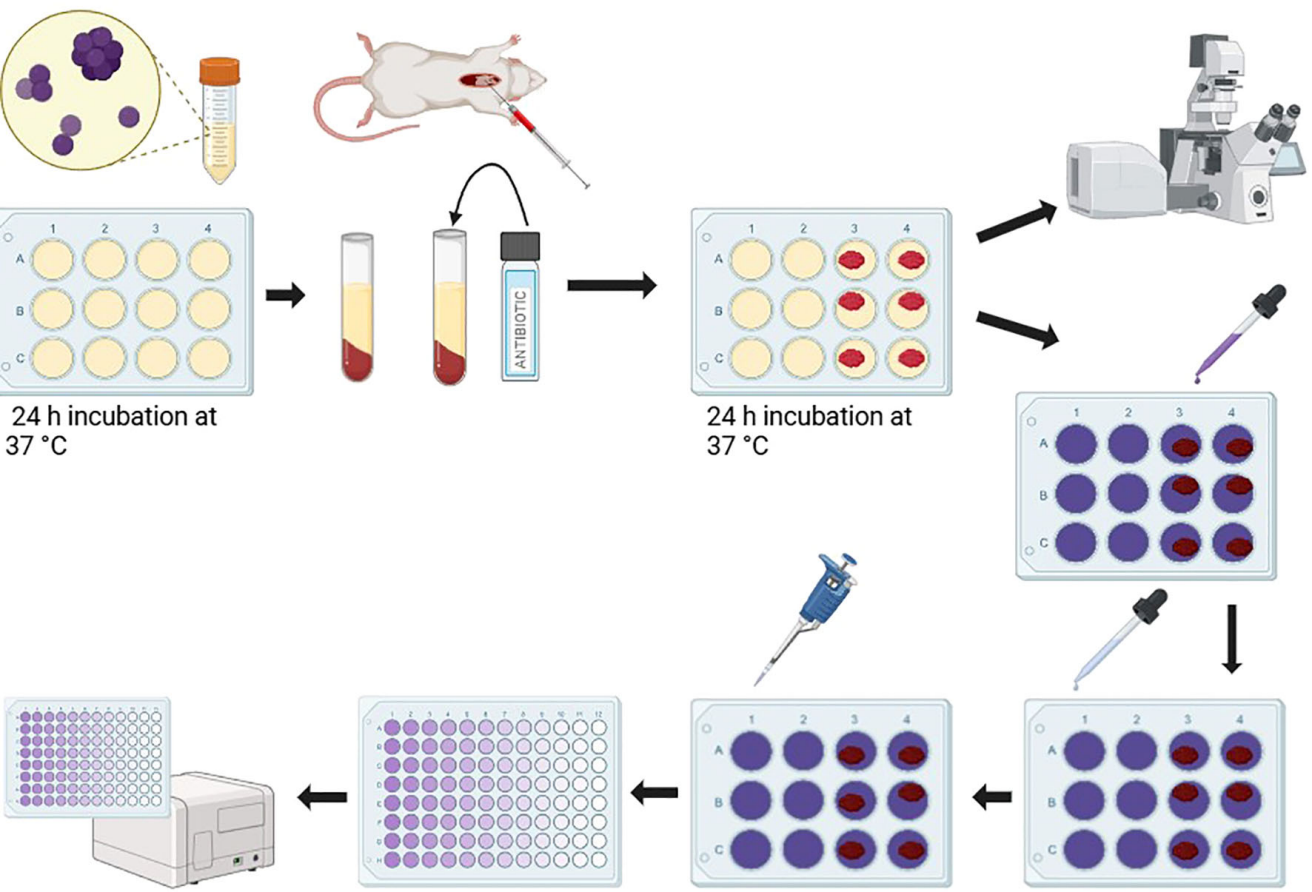
Schematic representation of experimental design.Figure generated with BioRender **^®^**.

Biofilm formation was assessed using a 12-well micro-titer plate model, adapted from the design in Kumar et al., 2019 as well as Bailey and Scott’s Diagnostic Microbiology, 14^th^ edition (Tille, 2017). Cells from an overnight culture were washed, counted, and resuspended to a final concentration of 1x10^6^ CFU/mL in LB. Aliquots of 1000 μL were used to seed the 12-well micro-titer plate (Corning Incorporated Costar). Plates were incubated for 24 h at 37°C and thrice washed with PBS to remove non-adherent cells (**Figure 1**).

### 2.3 Minimum bactericidal concentration and minimum biofilm eradication concentration

The Minimum Bactericidal Concentration (MBC) and Minimum Biofilm Eradication Concentration (MBEC) assays were adapted from Bailey and Scott’s Diagnostic Microbiology, 14^th^ edition (Tille, 2017) and the *In Vitro* MBEC Assay in Okae et al., 2022 to fit the parameters of this study. According to the European Committee for Antimicrobial Susceptibility Testing (EUCAST), the minimum bactericidal concentration is “the lowest concentration of an antibiotic that under defined *in vitro* conditions reduces by 99.9% the number of organisms in a medium containing a defined inoculum of bacteria within a defined period of time.” (European Committe for Antimicrobial Susceptibility Testing, 2000) The Minimum Biofilm Eradication Concentration can be defined as “the lowest concentration of antibiotic required to eradicate the biofilm (Ceri et al., 1999) or, in other words, the lowest concentration of antimicrobial agent that prevents visible growth in the recovery medium used to collect biofilm cells.” (Macia et al., 2014). Historically, these measures have been paired together because they are complementary, describing the concentration needed for complete cell death in planktonic and biofilm culture (Evans and Holmes, 1987). For the MBC, increasing concentrations of select antibiotics (gentamicin or vancomycin) were incubated with planktonic bacteria in a 12-well micro-titer plate for 24 h in a stagnant 37°C incubator. For gentamicin, the intervals ranged from 0 to 40 μg/mL (see supplemental 1). For vancomycin, the intervals ranged from 0 to 5 μg/mL. Wells were then thrice washed with PBS and the remaining bacteria was scrapped and resuspended in 3 mL of LB media, which was placed in a shaking 37°C incubator overnight. Following the bacterial recovery period, a colony forming unit (CFU) assay in the form of the drop plate method was performed, and 24 h later, colony formation was counted at the 10^-4^ dilution (Herigstad et al., 2001). For the MBEC, biofilms were formed as previously described over 24 h, were then thrice washed with PBS and treated with select antibiotics (gentamicin or vancomycin). For gentamicin, the intervals ranged from 0 to 10,000 μg/mL. For vancomycin, the intervals ranged from 0 to 1,000 μg/mL (see supplemental 1). After 24 h treatment, the MBEC was conducted in the same manner as the MBC. Each concentration for each condition was tested in triplicate.

### 2.4 Blood clot harvesting and preparation

All animal procedures were approved by the host institution’s Institutional Animal Care and Use Committee (IACUC protocol number 2020-0023). This protocol was adapted from Ku et al., 2024. C57 BL/6J mice (6–8 weeks of age; male) were anesthetized with 1–1.5% isoflurane. After reaching the surgical plane of anesthesia, approximately 300–500 µL of blood was removed via cardiac puncture. Animals were subsequently euthanized in accordance with institutional protocols. The blood was subsequently transferred to microcentrifuge tubes, where it was allowed to clot in the presence of LB media, LB media supplemented with vancomycin (20 µg/mL), or LB media supplemented with gentamycin (10 µg/mL). These blood clot preparations were subsequently applied to the biofilms, as outlined below.

### 2.5 Crystal violet assay

This assay was modeled after the protocol in O’Toole, 2011; Kumar et al., 2019, and Bailey and Scott’s Diagnostic Microbiology, 14th edition (Tille, 2017). Select wells were exposed to the experimental condition. The following conditions were tested: no treatment, 20 µg/mL gentamicin suspension, 10 µg/mL vancomycin suspension, gentamicin impregnated blood clot, vancomycin impregnated blood clot, and blood clot alone. Fresh LB media was replaced in each well such that the total volume remained 1000 µL. Plates were incubated for 24 h at 37°C, thrice washed with PBS to remove non-adherent cells, and stained with 0.1% crystal violet for 10 minutes. Biofilms were washed with sterile water to remove excess stain, then destained with 33% acetic acid (Fisher Scientific). The supernatant was transferred to empty wells and the absorbance was measured at 550 nm using a plate reader (BioTek EPOCH 2). The results were analyzed by Student’s t-test, comparing each transformant strain with its parent strain (**Figure 1**).

### 2.6 Metabolic assay

The following procedure was adapted from Haney et al., 2018 to fit the parameters of this study. Following a 24 h incubation of 24-well plates (Corning Incorporated Costar) in static conditions, media and planktonic cells were removed from their respective wells and replaced with the following treatments: control, gentamicin, vancomycin, blood clot, blood clot with gentamicin, and blood clot with vancomycin. In addition to the treatment, fresh LB Media containing 0.05% 2,3,5-Triphenyl Tetrazolium Chloride (TTC) (Carolina Biological Supply Company) was added to each well for a total volume of 1000 µL. Plates were then incubated at 37°C for 24 h. To remove used media and residual treatments, the biofilms were carefully washed three times with deionized water. To isolate the metabolized TTC, 500 µL of methanol was mixed gently in each well. To quantify the metabolized TTC, 200 µL from each well was aliquoted to a 96-well micro-titer plate (Wuxi NEST Biotechnology Co) and read on a plate reader (BioTek EPOCH 2) at an absorbance of 500 nm.

### 2.7 Architectural analysis

The following procedure was adapted from Haney et al., 2018 to fit the parameters of this study. Cells from an overnight culture were diluted to a final density of 0.005 at an absorbance reading of 600 nm, and 2 mL was seeded in MatTek 35 mm Glass Bottom Culture Dishes. After being incubated at 37°C for 24 h, media and unadhered cells were removed. Fresh media (2 mL) and treatments were added, as described above, centralized on the glass microwells of the dishes. After another 24 h incubation, dishes were washed twice with DI water. Care was taken to ensure the biofilms were not disrupted. The biofilms were centrally stained with 10.2μM SYTO^®^9 and 60μM propidium iodide diluted in DI water (FilmTracer^TM^ LIVE/DEAD^®^ Biofilm Viability Kit; Invitrogen Molecular Probes), protected from light, and allowed to sit for 20 minutes. The excess stain was removed and washed with DI water.

Samples were imaged using a Nikon A1R+ Confocal Microscope System equipped with a 60X objective, with analysis and 3D rendering performed using NIS-Elements C software. Bacterial quantification was performed with the open-source software ImageJ (version 1.54f, National Institutes of Health, USA) by Fiji (Schindelin et al., 2012). Specifically, the live and dead percentages were determined using the MorphoLibJ plugin and Biofilm Viability Checker macro (Mountcastle et al., 2021). Imaging was performed in biological triplicates. Imaging data were generated in the Flow Cytometry and Imaging Core at Western Michigan University Homer Stryker M.D. School of Medicine (**Figure 1**).

### 2.8 Statistical analysis

Data are expressed as mean ± SD and n represents the number of discrete samples. Paired t-tests were used to compare the effect of a given treatment condition on A_500_. GraphPad Prism 10.2.2 was used for data analysis and figure generation.

## 3 Results

### 3.1 Crystal violet assay

**Figure 2:**
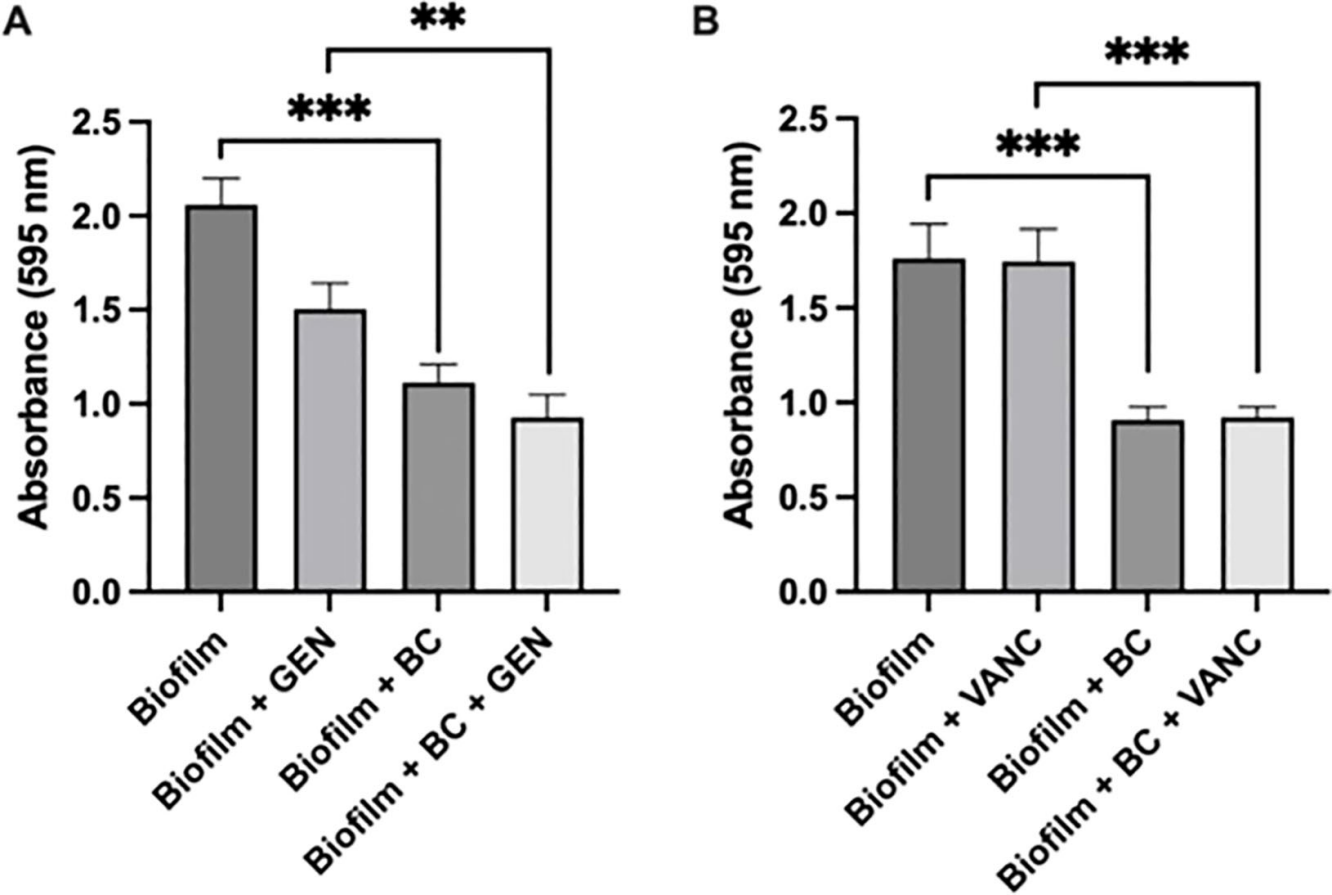
Biofilm formation and Crystal Violet Assay. Crystal Violet assay measuring *Staphylococcus epidermidis* biofilm formation following exposure to blood clot, gentamicin **(A)**, vancomycin **(B)**, or their combination. Significance indicated by ** (p<0.0032) and **** (p<0.0008). Each experiment was performed in triplicate. Gentamicin trials were conducted across 6 independent experiments, while vancomycin trials were conducted across 3 independent experiments.

To examine the impact of our blood clot/antibiotic system on biofilm density, we formed *S. epidermidis* biofilms and then exposed them to antibiotics suspended in media vs blood clots impregnated with an equal concentration of antibiotics. A blood clot alone condition and an untreated condition served as controls. We selected two antibiotics with differing mechanisms of action to test, vancomycin and gentamicin. In clinical settings, vancomycin, a glycopeptide, is often selected as the first line option against *S. epidermidis* infections due to the frequency of methicillin resistance (Lee and Anjum, 2024). Gentamicin, an aminoglycoside, has been proposed as an alternative option (Chambers and Pallagrosi, 1973) (Karmakar et al., 2016). Our experiments showed that after 24 h, there was a significant reduction in biofilm density after blood clot/antibiotic exposure compared to antibiotic alone (**Figure 2**). There was approximately a 50% reduction in density between the biofilm control group and the biofilm with blood clot, with a p value less than 0.0008. Between the biofilm with gentamicin condition and the biofilm with gentamicin and blood clot condition, the reduction was approximately 33%, with a p value less than 0.0032. For the biofilm with vancomycin condition compared to the biofilm with vancomycin and blood clot, the reduction was approximately 40%, with a p value less than 0.0008. This trend, being evident across both antibiotics tested, suggests that the blood clot facilitates antibiotic susceptibility, potentially due to increased penetration. Additionally, exposure to liquid antibiotics alone caused a significant increase in biofilm density compared to the untreated control. This aligns with the current literature, which suggests that exposing biofilms to subtherapeutic concentrations of antibiotics elicits a stress response, subsequently increasing ECM production (Lories et al., 2020). Surprisingly, we observed that exposure to the blood clot alone caused a reduction in biofilm density. This reduction was not statistically different than the effect of the blood clot/antibiotic combination, suggesting that the mechanism of effect is dependent on the blood clot, and not the antibiotic. Elucidating the mechanism behind this requires further investigation. We hypothesize that select enzymes within the blood clot may play a role in breaking down the ECM. One potential enzyme is matrix metalloprotease 1 (MMP-1) which has already been shown to disrupt *Enterococcus* biofilms (Kumar et al., 2019).

### 3.2 Metabolic changes

**Figure 3:**
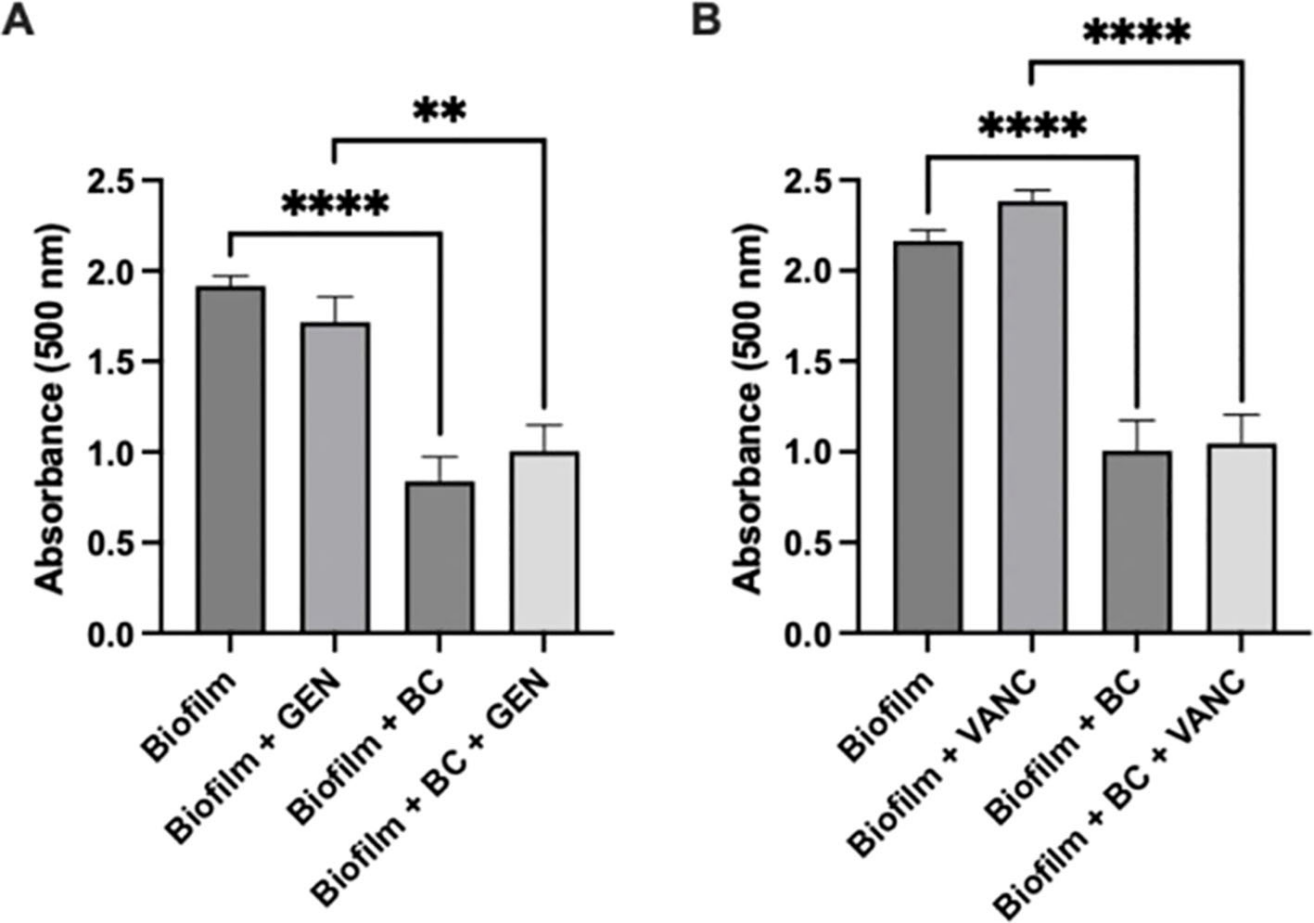
Metabolic Assay. Tetrazolium chloride assay of *Staphylococcus epidermidis* biofilm viability following exposure to blood clot, gentamicin **(A)**, vancomycin **(B)**, or their combination. Statistical significance indicated by ** (p<0.0032) and **** (p<0.0001). Each experiment was performed in triplicate, with a total of 2 independent experiments conducted.

To determine the impact of the blood clot/antibiotic system on the metabolic activity of the biofilm, we repeated the experiment using a TTC assay. The conditions and antibiotics tested during the Crystal Violet assay were maintained. Our results mirrored the results of the crystal violet assay (**Figure 3**). There was approximately a 55% reduction in density between the biofilm control group and the biofilm with blood clot, with a p value less than 0.0001. Between the biofilm with gentamicin condition and the biofilm with gentamicin and blood clot condition, the reduction was approximately 40%, with a p value less than 0.0032. For the biofilm with vancomycin condition compared to the biofilm with vancomycin and blood clot, the reduction was approximately 55%, with a p value less than 0.0001. After 24 h, there was a significant reduction in the metabolic activity of the biofilm after exposure to the blood clot/antibiotic and blood clot alone compared to the untreated control and antibiotic alone. Once more, the biofilms treated with antibiotics displayed an increase in metabolic activity.

### 3.3 Alterations in biofilm architecture

Recognizing the density and metabolic differences between treatments, we sought to visually confirm these relations through architectural imaging. Such imaging was performed via confocal microscopy, a critical technology used to determine specific relations not otherwise discovered within a biofilm. Confocal microscopy non-invasively visualizes the overall components and individual bacteria within a fully hydrated biofilm (Pitts and Stewart, 2008; Palmer et al., 2006). Additionally, the 3D capabilities through z-plane imaging allows for visualization of spatial positioning and treatment penetration into the biofilm (Reichhardt and Parsek, 2019).

**Figure 4:**
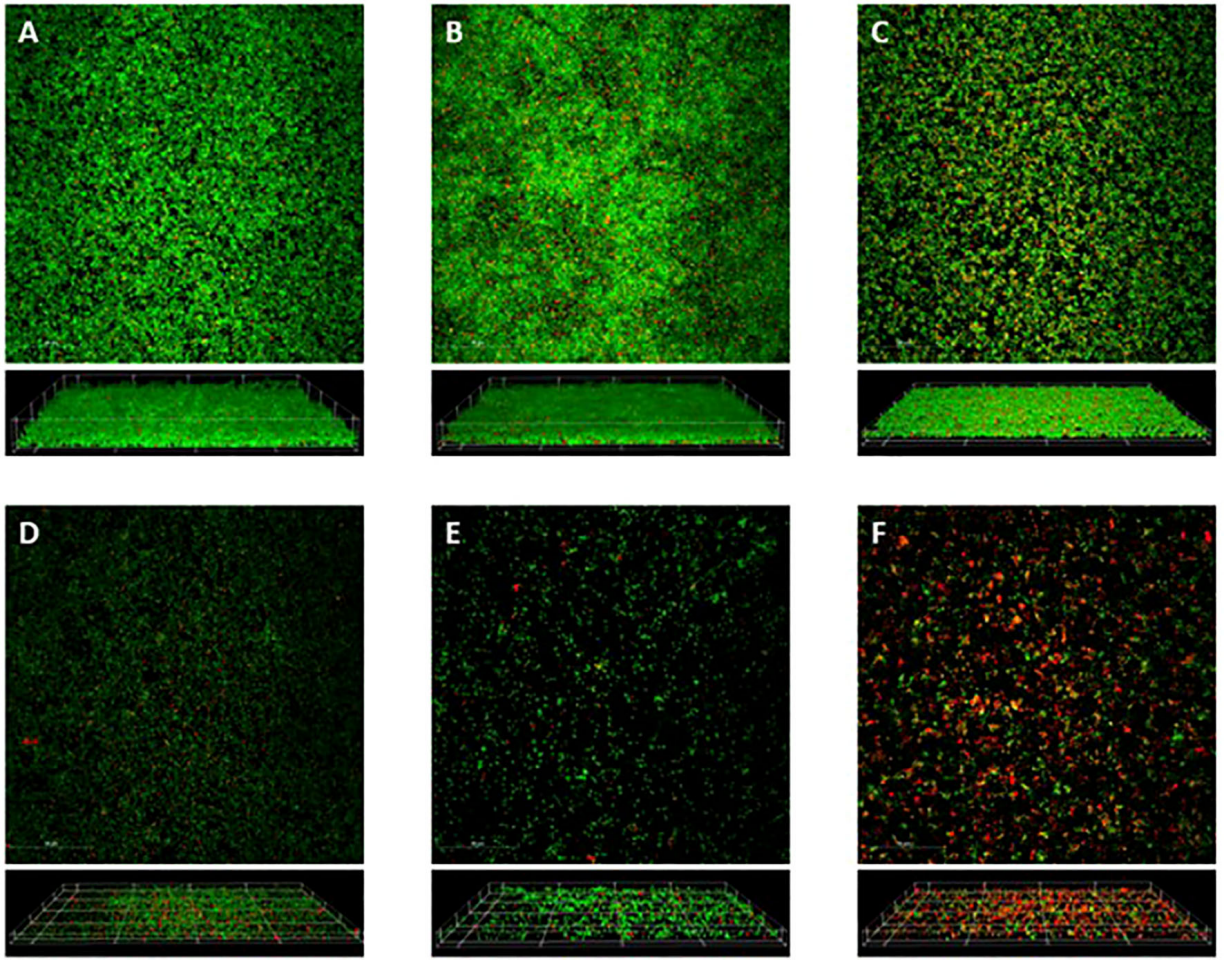
Confocal Microscopy. Confocal microscopy of 24 h old *S. epidermidis* biofilms formed on glass bottom culture dishes when either left untreated **(A)**, or treated with vancomycin (20 µg/mL) **(B)**, gentamicin (10 µg/mL) **(C)**, blood clot **(D)**, blood clot impregnated with vancomycin (20 µg/mL) **(E)**, or blood clot impregnated with gentamicin (10 µg/mL) **(F)**. Following 24 h treatments, biofilms were stained with SYTO-9 (green=live) and propidium iodide (red=dead). Image stacks were acquired along the z-plane using a Nikon A1R+ Confocal Microscope System. Top-down maximum projections (top panels) and 3D reconstructions (bottom panels) were made using NIS-Elements C software. The scale bars in the top panels represent 50 µm.

Utilizing the same 24 h treatments as above, the biofilms were stained with a Live/Dead stain, SYTO^®^9 and propidium iodide, then imaged in multiple slices along the z-plane and visualized in both a maximum intensity projection and a 3D rendering (**Figure 4**). A portrayal of a *S. epidermidis* biofilm is seen within the control condition, as it creates a cohesive sheet of bacteria (**Figure 4A**). The vancomycin treatment exhibits an increased abundance of bacteria compared to the control condition (**Figure 4B**). In contrast to the vancomycin, the gentamicin treatment appears to create a slightly dispersed biofilm, consisting of a more scattered array of clumps and planktonic bacteria (**Figure 4C**). This relation between antibiotic treatments is supported previously in the densities of each condition.

Meanwhile, the biofilms treated with blood clots (**Figures 4D-F**), exhibit a noticeable visual decrease of bacteria compared to the bacterial sheets seen in conditions without blood clots. Rather than the cohesive bacterial unit, these conditions have greater separation between colonies, with multiple areas absent of bacteria. Moreover, within the blood clot treatments, there appears to be less bacteria in the conditions where blood clot was impregnated with antibiotic (**Figures 4E, F**), suggesting an amplified effect when these treatments are used together.

**Figure 5:**
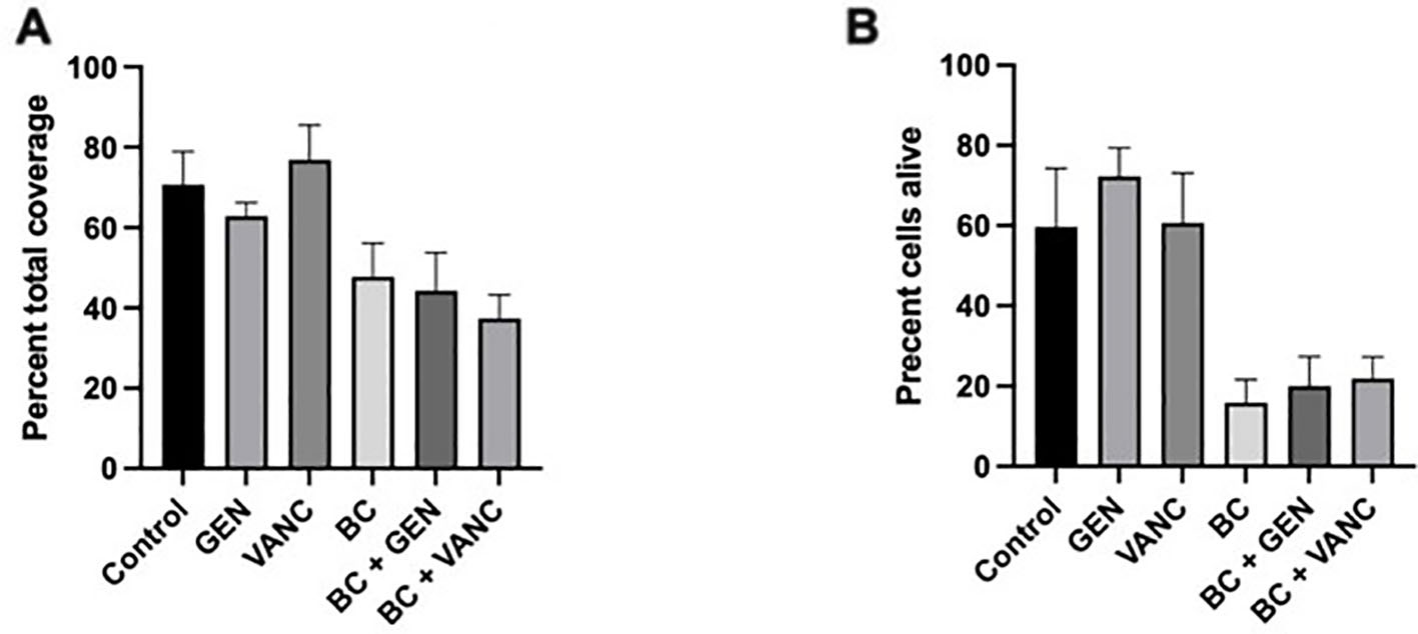
Confocal Analysis of Biofilm Coverage. Live-dead staining of *Staphylococcus epidermidis* biofilms without treatment (control) and following administration of gentamicin (GEN) and vancomycin (VANC), alone and conjugated with a blood clot (bc). **(A)** Depicts percentage of total bacterial coverage, and **(B)** depicts percentage of living cells. Analysis performed using Prism. Each experiment was preformed in triplicate, with a total of 3 independent experiments conducted.

This visual relation of treatment type to bacteria coverage is further supported through the quantification of these images, with the open-source domain ImageJ by Fiji and the Biofilm Viability Checker (Mountcastle et al., 2021; Schindelin et al., 2012). The percentage of the visual plane covered with bacteria, live or dead, appears greatly dependent on the presence of blood clot (**Figure 5A**). The percentages for total bacterial coverage for no treatment range from 55.5% to 84.1%, for gentamicin values range from 58.5% to 69.4%, for vancomycin values range from 49.5% to 86.9%, for blood clot values range from 31.4% to 57.8%, for blood clot with gentamicin values range from 27.1% to 59.8%, and for blood clot with vancomycin values range from 28.9% to 48.7%. The addition of a blood clot appears to decrease the bacterial coverage by an average of a third to that of the non-blood clot condition. Additionally, the biofilms treated with blood clot appear to be comprised of more dead cells throughout (**Figure 4**). This is supported in the quantification of the biofilm viability, in which more propidium iodide staining is present in the biofilms treated with blood clots (**Figure 5B**). Thus, the combination of a dispersed biofilm, increased death, and planktonic form visualization throughout the biofilms treated with blood clot, and to a greater degree when impregnated with antibiotic, suggests its effectiveness as a viable treatment. The percentages for alive cells for no treatment range from 32.3% to 78.7%, for gentamicin the values range from 58.8% to 83.1%, for vancomycin the values range from 41.3% to 83.9%, for blood clot the values range from 5.8% to 35.7%, for blood clot with gentamicin the values range from 5.6% to 29.2%, for blood clot with vancomycin the values range from 11.1% to 36.0%.

Qualitatively, the presence of a 24 h blood clot treatment alone or impregnated with antibiotics appears to decrease bacteria abundance. This trend warrants further testing and investigation as despite the appearance, there is no significant difference in total bacterial coverage between samples. The p-values for total bacterial coverage are as follows: control to gentamicin p=0.98, control to vancomycin p=0.99, blood clot to control p=0.43, blood clot with gentamicin to control p=0.29, blood clot with vancomycin to control p=0.12, blood clot to gentamicin p=0.78, blood clot to vancomycin p=0.32, blood clot to blood clot with gentamicin p=0.99, blood clot to blood clot with vancomycin p=0.94, blood clot with gentamicin to gentamicin p=0.62, and blood clot with vancomycin to vancomycin p=0.08. When looking at the percentage of alive cells, there are two significant p-values (blood clot to gentamicin and blood clot with gentamicin to gentamicin), however the rest have no significant differences. The p-values for percent of alive cells are as follows: blood clot to gentamicin p=0.02, blood clot with gentamicin to gentamicin p=0.03, control to gentamicin p=0.73, control to vancomycin p=0.99, blood clot to control p=0.22, blood clot with gentamicin to control p=0.24, blood clot with vancomycin to control p=0.39, blood clot to vancomycin p=0.09, blood clot to blood clot with gentamicin p=1.0, blood clot to blood clot with vancomycin p=0.99, and blood clot with vancomycin to vancomycin p=0.18.

The variations between visual appearance and quantification may be due to the multiple limitations with quantifying Live/Dead staining of biofilms through confocal microscopy. These include thickness and density of biofilm, flatness of biofilm, and background staining (Mountcastle et al., 2021). Additional limitations occur with the binding of propidium iodide to biofilm extracellular matrices and extracellular DNA which in turn can overestimate dead cell counts (Rosenberg et al., 2019). In response to these limitations, the primary use of confocal microscopy in biofilm analysis is to demonstrate qualitative results through biofilm visualization (Mountcastle et al., 2021). Thus, despite the limitations and the variation in quantifying the confocal microscopy images, the qualitative trends express a promising decrease in bacterial abundance and viability in the presence of blood clot treatment.

To further determine the quantifiable and mechanistic properties of the blood clot treatment, we recommend further comprehensive procedures. This includes the utilization of fluorescently tagged *S. epidermidis* combined with time-lapse imaging to allow for the visualization of treatment penetration and biofilm degradation in real time (Palmer et al., 2006) (Schlafer and Meyer, 2017). Additionally, to determine individual bacteria viability throughout the biofilm and in relation to the placement of the blood clot, we propose the use of flow cytometry (Servain-Viel et al., 2024).

## 4 Discussion and conclusion

Our study shows that fresh fibrin blood clots offer a promising option for the treatment of *S. epidermidis* biofilms and have the potential to prevent infections. Specifically, we saw that the use of blood clot treatments reduces cell load and disrupts architecture. As the U.S population ages, the number of patients with prosthetic implants susceptible to these infections will continue to rise (Fischbacher and Borens, 2019). Autologous blood clots potentially serve as a cost effective, immunologically inert option for treating these highly resistant infections. It is therefore necessary to continue exploring this treatment through multiple avenues. Primarily, further exploration of the intrinsic mechanism of the blood clot is warranted. To start, continued confocal imaging analysis especially through bacterial staining percentages will foster a mechanistic hypothesis. From there, to identify a more comprehensive understanding of the treatment impact on biofilm structure and environment we will employ scanning electron microscopy (Alhede et al., 2012). Further, a novel approach would be to utilize single-cell RNA sequencing to identify the transcriptional stress response of the bacteria to the blood clot treatment (Korshoj and Kielian, 2024). Once a mechanistic pathway is determined, we suggest further identification of blood clot characteristics, including the blood clot proximity to the biofilm using transwell inserts, and the effects of individual blood clot components, specifically MMP (Kumar et al., 2019). We also suggest further investigation into the relationship between antibiotics and blood clots in suspension, including potential binding mechanisms. Other investigations have suggested certain antibiotics are capable of binding individual components of blood clots, such as fibrin. Recently, fibrin-based nanoparticles have been used to bind and deliver the antibiotic vancomycin into *Staphylococcus aureus* biofilms (Scull et al., 2024). Platelet rich fibrin has also been used as a delivery mechanism for the antibiotic’s vancomycin, linezolid, and gentamicin in an oral surgery context, although specific binding mechanism was not ascertained. They found that vancomycin interfered with PRF formation. Gentamicin and linezolid did not change the physical properties of PRF and were released from membranes in the time intervals examined (Bennardo et al., 2023). It is likely that unique properties of the different antibiotics contribute to the viability of molecular binding, if it is occurring, and therefore we encourage further exploration of these models.

Additionally, it is known that biofilm susceptibility to treatments is impacted by its developmental stage, thus we suggest investigating the addition of blood clot treatments at various biofilm stages (Wang et al., 2023). Through these continued studies, the specificities and mechanism of the blood clot treatment will be established, and additional, translational studies can be focused on. Imperatively, this treatment needs to be investigated using animal models, with and without antibiotics. It would also be beneficial to understand if treatment improves the effectiveness of consequent IV antibiotic treatment, reducing the need for surgical debridement. Finally, we recommend exploring the impact that this treatment can have on polyclonal biofilms, particularly in other clinical models including chronic wounds and burns. Ultimately, the encouraging results found within this study promote a wide range of prospective investigations utilizing blood clots to eradicate biofilms.

## Data Availability

The raw data supporting the conclusions of this article will be made available by the authors, without undue reservation.
