# Supplementary material for: A novel strategy for eradication of staphylococcal biofilms using blood clots
Source: Front Cell Infect Microbiol. 2025 Jan 31;15:1507486. doi: 10.3389/fcimb.2025.1507486 (PMC11827428; doi:10.3389/fcimb.2025.1507486)
Supplement: Supplementary file 2 [file Table2.docx]

Supplementary Material

**Minimum Bactericidal Concentration Assay.** Analysis of inhibition of a 24 h old *Staphylococcus epidermidis* liquid culture after exposure to various concentrations of A. gentamicin, B. vancomycin. Serial dilution preformed and plated on LB agar. Minimum bactericidal concentration is estimated to be at 10 μ/mL for both antibiotics.

| A. Minimum Bactericidal Concentration for Gentamicin | | |  | |  | |  | |  | |  | |  | |  |  |
| --- | --- | --- | --- | --- | --- | --- | --- | --- | --- | --- | --- | --- | --- | --- | --- | --- |
| \| µg/mL \| **0** \| **0.05** \| **0.1** \| **0.2** \| **0.5** \| **0.75** \| **1** \| **2** \| **5** \| **10** \| **20** \| **40** \| \| --- \| --- \| --- \| --- \| --- \| --- \| --- \| --- \| --- \| --- \| --- \| --- \| --- \| \| Experiment 1 \| TNTC \| TNTC \| TNTC \| TNTC \| TNTC \| TNTC \| TNTC \| TNTC \| TNTC \| 0 \| 0 \| 0 \| \| Experiment 2 \| TNTC \| TNTC \| TNTC \| TNTC \| TNTC \| TNTC \| TNTC \| TNTC \| 54 \| 0 \| 0 \| 0 \| \| Experiment 3 \| TNTC \| TNTC \| TNTC \| TNTC \| TNTC \| TNTC \| TNTC \| TNTC \| TNTC \| 0 \| 0 \| 0 \| |  |  | |  | |  | |  | |  | |  | |  |  |  |
|  |  |  | |  | |  | |  | |  | |  | |  |  |  |
| B. Minimum Bactericidal Concentration for Vancomycin | | |  |  |  |  |  |  |  |  |  |  |  |  |  |  |
| \| µg/mL \| **0** \| **0.05** \| **0.1** \| **0.2** \| **0.5** \| **0.75** \| **1** \| **2** \| **5** \| **10** \| **20** \| **40** \| \| --- \| --- \| --- \| --- \| --- \| --- \| --- \| --- \| --- \| --- \| --- \| --- \| --- \| \| Experiment 1 \| TNTC \| TNTC \| TNTC \| TNTC \| TNTC \| TNTC \| TNTC \| TNTC \| TNTC \| 0 \| 0 \| 0 \| \| Experiment 2 \| TNTC \| TNTC \| TNTC \| TNTC \| TNTC \| TNTC \| TNTC \| TNTC \| TNTC \| 0 \| 0 \| 0 \| \| Experiment 3 \| TNTC \| TNTC \| TNTC \| TNTC \| TNTC \| TNTC \| TNTC \| TNTC \| TNTC \| 0 \| 0 \| 0 \| |  |  | |  |  |  |  |  |  |  |  |  |  |  |  |  |

TNTC = too numerous to count

**Minimum Biofilm Eradication Concentration Assay.** Analysis of inhibition of a 24 h old *Staphylococcus epidermidis* biofilm after exposure to various concentrations of A. gentamicin, B. vancomycin. Serial dilution preformed and plated on LB agar. Minimum biofilm eradication concentration is estimated to be between 200 and 500 μ/mL or gentamicin and between 300 and 500 μ/mL vancomycin.

A. Minimum Biofilm Eradication Concentration for Gentamicin

| µg/mL | **0** | **10** | **30** | **50** | **70** | **100** | **150** | **200** | **500** | **1000** | **5,000** | **10,000** |  |  |
| --- | --- | --- | --- | --- | --- | --- | --- | --- | --- | --- | --- | --- | --- | --- |
| Experiment 1 | TNTC | TNTC | TNTC | TNTC | TNTC | TNTC | TNTC | TNTC | 0 | 0 | 0 | 0 |  |  |
| Experiment 2 | TNTC | TNTC | TNTC | TNTC | TNTC | TNTC | TNTC | TNTC | 0 | 0 | 0 | 0 |  |  |
| Experiment 3 | TNTC | TNTC | TNTC | TNTC | TNTC | TNTC | TNTC | TNTC | 0 | 0 | 0 | 0 |  |  |
| B. Minimum Biofilm Eradication Concentration for Vancomycin | | | | | | | | | | | | | | |
| \| µg/mL \| **None** \| **20** \| **40** \| **60** \| **100** \| **150** \| **200** \| **250** \| **300** \| **500** \| **750** \| **1,000** \| \| --- \| --- \| --- \| --- \| --- \| --- \| --- \| --- \| --- \| --- \| --- \| --- \| --- \| \| Experiment 1 \| TNTC \| TNTC \| TNTC \| TNTC \| TNTC \| TNTC \| TNTC \| 1 \| 9 \| 0 \| 0 \| 0 \| \| Experiment 2 \| TNTC \| TNTC \| TNTC \| TNTC \| TNTC \| TNTC \| TNTC \| 2 \| 0 \| 0 \| 0 \| 0 \| \| Experiment 3 \| TNTC \| TNTC \| TNTC \| TNTC \| TNTC \| TNTC \| TNTC \| 2 \| 0 \| 0 \| 0 \| 0 \| | | | | | | | | | | | | | | |

|  | | | | | | | |  | | |  |  |  |  |  |  |  |  |
| --- | --- | --- | --- | --- | --- | --- | --- | --- | --- | --- | --- | --- | --- | --- | --- | --- | --- | --- |
|  | | | | | | | |  | | |  |  |  |  |  |  |  |  |
|  | | | | | | | |  | | |  |  |  |  |  |  |  |  |
|  |  |  |  |  |  |  |  |  |  |  |  |  |  |  |  |  |  |  |
|  |  |  |  |  |  |  |  | |  |  |  |  |  |  |  |  |  |  |
|  | | | | | | | |  | | |  |  |  |  |  |  |  |  |
|  | | | | | | | |  | | |  |  |  |  |  |  |  |  |
|  | | | | | | | |  | | |  |  |  |  |  |  |  |  |
